# Comparing estimates of household expenditures between pictorial diaries and surveys in three low- and middle-income countries

**DOI:** 10.1371/journal.pgph.0001739

**Published:** 2023-04-04

**Authors:** Adrianna Murphy, Benjamin Palafox, Jephat Chifamba, Iolanthé M. Kruger, Brian J. Ncube, Tatenda L. Ncube, Sumathy Rangarajan, Elizabeth Catherina Swart, Lungiswa Tsolkile, Marjan Walli-Attaei, Nicola J. West, Karen E. Yeates, Salim Yusuf, Martin McKee, Kara Hanson

**Affiliations:** 1 Department of Health Services Research and Policy, London School of Hygiene & Tropical Medicine, London, United Kingdom; 2 Department of Biomedical Sciences, University of Zimbabwe, Harare, Zimbabwe; 3 Africa Unit for Transdisciplinary Health Research (AUTHeR), North-West University, Potchefstroom, South Africa; 4 Population Health Research Institute, McMaster University and Hamilton Health Sciences, Hamilton, Canada; 5 Department of Dietetics and Nutrition, University of the Western Cape, Cape Town, South Africa; 6 School of Public Health, University of the Western Cape, Cape Town, South Africa; 7 Pamoja Tunaweza Women’s Centre, Moshi, Tanzania; 8 Department of Medicine, Queen’s University, Kingston, Canada; 9 Department of Global Health & Development, London School of Hygiene & Tropical Medicine, London, United Kingdom; Northeastern University, UNITED STATES

## Abstract

In most low- and middle-income countries (LMICs), household out-of-pocket (OOP) health spending constitutes a major source of healthcare financing. Household surveys are commonly used to monitor OOP health spending, but are prone to recall bias and unable to capture seasonal variation, and may underestimate expenditure–particularly among households with long-term chronic health conditions. Household expenditure diaries have been developed as an alternative to overcome the limitations of surveys, and pictorial diaries have been proposed where literacy levels may render traditional diary approaches inappropriate. This study compares estimates for general household and chronic healthcare expenditure in South Africa, Tanzania and Zimbabwe derived using survey and pictorial diary approaches. We selected a random sub-sample of 900 households across urban and rural communities participating in the Prospective Urban and Rural Epidemiology study. For a range of general and health-specific categories, OOP expenditure estimates use cross-sectional survey data collected via standardised questionnaire, and data from these same households collected via two-week pictorial diaries repeated four times over 2016–2019. In all countries, average monthly per capita expenditure on food, non-food/non-health items, health, and consequently, total household expenditure reported by pictorial diaries was consistently higher than that reported by surveys (each p<0.001). Differences were greatest for health expenditure. The share of total household expenditure allocated to health also differed by method, accounting for 2% in each country when using survey data, and from 8–20% when using diary data. Our findings suggest that the choice of data collection method may have significant implications for estimating OOP health spending and the burden it places on households. Despite several practical challenges to their implementation, pictorial diaries offer a method to assess potential bias in surveys or triangulate data from multiple sources. We offer some practical guidance when considering the use of pictorial diaries for estimating household expenditure.

## Introduction

There is increasing interest in monitoring national health expenditure from different financing sources to inform health system resource planning and allocation, and increase efficiency. This interest has resulted in several guidelines on how best to measure public and private health expenditure [1,2]. The most challenging component is the measurement of household out-of-pocket (OOP) health expenditure. In most low- and middle-income countries (LMICs), private expenditure overall accounts for 25–60% of total expenditure on health. While private expenditure can come from private firms or enterprises, private insurance schemes, non-governmental organisations or households, in LMICs it consists primarily of household OOP expenditure. Household OOP expenditure is the largest source of healthcare financing in low-, and lower middle-income countries. In upper middle-income countries it is the second largest, after government transfers, but still occupies almost 40% of expenditures [3].

The most frequently used method of capturing household healthcare utilisation and OOP expenditure is household surveys [4,5]. However, household surveys have various problems. First, because they ask about what happened over some period in the past, they depend on accurate recall by the subjects [4,6,7]. To minimise the risk of recall bias, those designing the surveys typically keep the recall period short, often as little as four weeks. However, people with chronic health conditions, who often have more than one, require long-term and continuous interactions with the health system. These interactions may be brief and individually inexpensive but, cumulatively, they can impose a substantial burden [8]. Surveys that use a recall period for outpatient visits of more than 2–3 days can result in significant forgetting of events, and the number of events forgotten increases proportionately with the length of the recall period [9,10].

Another problem is that household surveys, often of necessity given the need to transport fieldworkers into communities, may draw their samples at a particular time of year–despite considerable evidence that both general household consumption and healthcare utilisation are highly seasonal, especially in agrarian societies [4,7,11]. Taken together, these issues may result in the under-reporting of health care expenditure in surveys. Research from rural Ghana, for example, showed that household surveys may underestimate morbidity and healthcare utilisation by up to a third and that the vulnerability of survey estimates to bias may vary by the financial status of the household [5].

One way to overcome the limitations inherent in household surveys is collection of longitudinal data, and triangulation of these data with other data sources (e.g. surveys, administrative data), recognising each source’s inherent biases and limitations [2]. An alternative means of collecting household OOP health expenditure that addresses the limitations of surveys and may help to understand the extent of bias in surveys is a household expenditure diary. Such diaries, considered the most accurate method of recording daily food consumption and expenditure [12], are now being adopted in health research. Diaries have the advantage of reducing the recall bias associated with self-reported survey data as expenditure is recorded as it occurs [4]. However, methodological research conducted in Tanzania suggests that such gains could be outweighed if traditional diaries are used in settings with low literacy, potentially excluding some of the most vulnerable individuals, especially in rural areas [7].

One way of overcoming this is to use pictorial diaries. Examples of pictorial diaries to collect household health and general expenditure include research conducted in Nigeria [13], Tanzania and Gambia [14], and they have been used to capture health care utilisation data in Ghana [5]. These studies demonstrate the feasibility of pictorial diaries for health expenditure research. However, the reports of experience of using them are few, and in particular, there is little on how to ensure they are implemented effectively. To our knowledge, only one study using pictorial diaries in Tanzania and Gambia has provided reflection on practical considerations related to their implementation [14]. This study identified important methodological issues that must be addressed, including for how long a diary should be maintained and who in a household should be responsible for recording information in the diary, a complex issue given the challenges of understanding intra-household allocation of decision-making for health spending [15].

Moreover, no study has compared the expenditure estimates produced by pictorial diaries with survey estimates from the same population. This is important because such a comparison is needed to determine the validity of these methods, their relative strengths and weaknesses, and their appropriateness for collecting health expenditure data in populations in low-resource settings. Using a sub-sample of households from the Prospective Urban and Rural Epidemiology (PURE) study, we aimed to compare general household and chronic health care expenditure estimates derived from a survey and diaries, and to consider the implications of our findings for the choice of method in future health expenditure research. We also add to the literature on implementing these studies in settings that present challenges regarding regular access for data collection.

## Materials and methods

### PURE study household survey

The PURE study [16] is a large ongoing international cohort study of non-communicable disease incidence, mortality and risk factors in individuals from urban and rural communities in 21 countries, with health expenditure data available from 18 countries: Canada, Sweden, Brazil, Chile, Malaysia, Poland, South Africa, Turkey, China, the Philippines, Colombia, Iran, the Occupied Palestinian Territory, Bangladesh, India, Pakistan, Zimbabwe and Tanzania. Data collection in PURE has been described in detail elsewhere [16]. Briefly, in each country, communities were selected to achieve a mix of rural and urban populations while ensuring the feasibility of data collection (e.g. processing blood samples) and long-term follow-up. Households were selected to represent the sociodemographic composition of communities, and the sociodemographic characteristics and death rates of the samples of the first 17 participating countries have been found to be similar to their respective national populations [17]. Further details on the representativeness of the PURE cohort are included in S1 Appendix.

Within each selected household, all individuals aged 35–70 years were eligible to participate. Baseline data collection occurred between 2005 and 2014, depending on when the country joined the PURE Study; information on sociodemographic characteristics and health status were collected using a standardised questionnaire. The round of follow-up data collection that included out-of-pocket health expenditures began in 2014, and the year in which follow-up data were completed in each country is shown in S2 Appendix. The health expenditure questions are included in S3 Appendix. For this study, we used PURE household expenditure data from South Africa, Tanzania and Zimbabwe.

### Pictorial diaries

**Fig 1:**
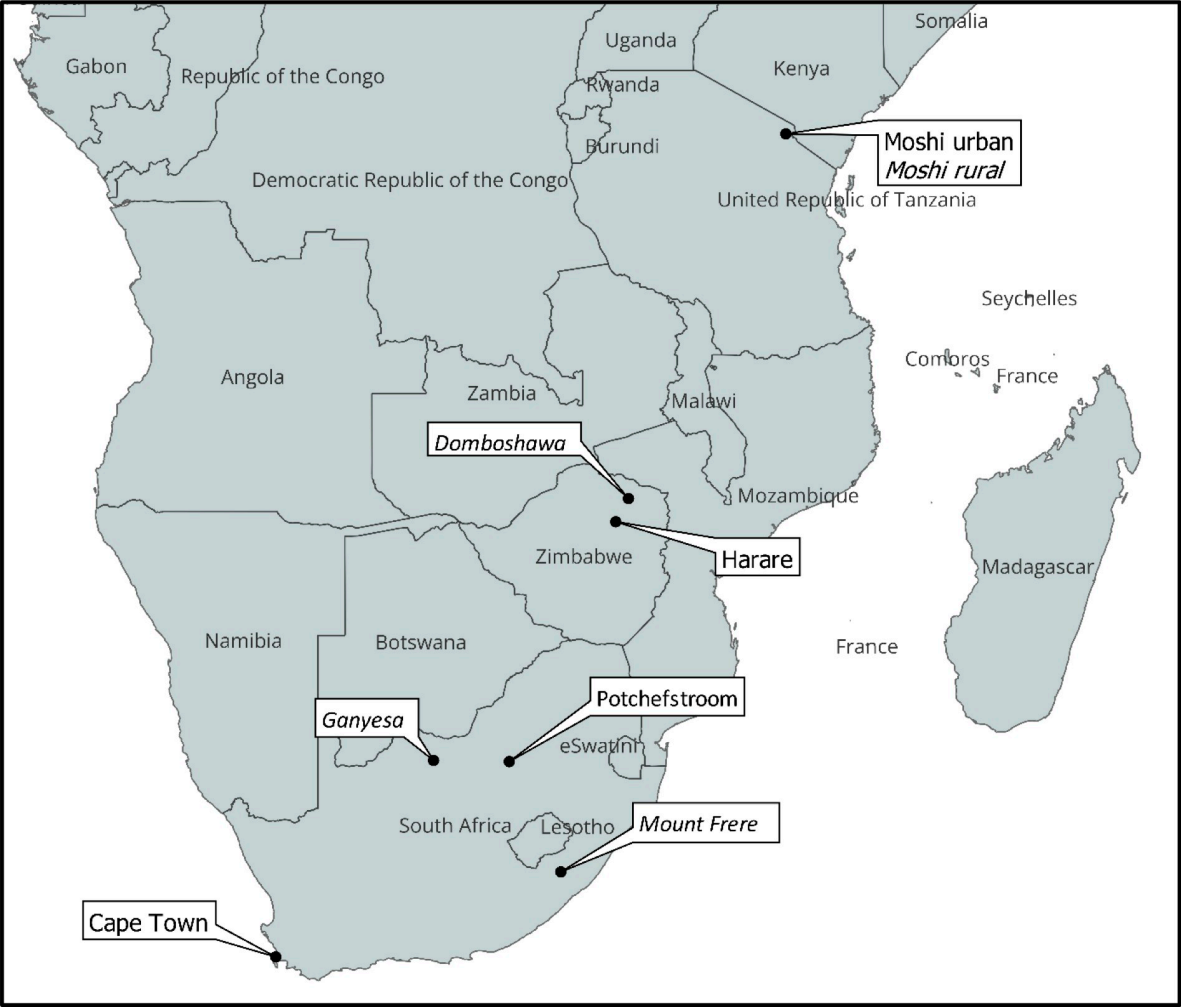
Map of study locations. Rural sites in italics. Source: https://www.naturalearthdata.com/downloads/10m-cultural-vectors/. Licence: CC0 1.0 Universal (CC0 1.0) Public Domain Dedication.

We randomly selected subjects from different households from the list of PURE study participants and asked them to complete pictorial diaries for their household. This study included all of the communities involved in the PURE study shown in Fig 1. In each of the three countries, we aimed to recruit 300 participants divided equally across the number of included communities (i.e. 75 participants from each of the 4 South African communities; 150 from each of the 2 included communities in Tanzania and Zimbabwe). Sample size was determined pragmatically, based on what was deemed feasible by the research team within the time and resources available.

Participants were invited to complete a pictorial daily expenditure diary over a two-week (14-day) period, repeated four times throughout the study (between September 2016 and January 2019). The diaries included pictures and text cues for participants to report daily expenditure on general household items and all healthcare-related expenses. As this study was designed to capture expenditure related to chronic conditions, our diaries focused on care received in the outpatient sector. The categories included in the diaries were determined by referring to commonly used survey tools and consulting local research partners and study participants on contextually appropriate lists of expenditure categories. As a result, our diaries included some categories not typically included in expenditure surveys. The categories included in the diary were: food inside the home, food outside the home, tobacco and alcohol, rent, education, durables, clothing, transportation, family transfers, farm and garden items, church donations, outpatient consultations, medicines, transportation to health facilities, alternative/traditional consultation and medicine, caregiver, religious healing, lost day’s wages due to being ill, lost day’s wages due to seeking medical care, and other categories. Expenditures reimbursed by health insurance were excluded. The pictorial diaries were piloted with 2–3 participants in each country and revised according to feedback. The final pictorial diary tool used in Tanzania is included in S4 Appendix as an example.

Field workers visited each participant’s household on day one of the data collection periods to provide instruction on how to complete the diary. Diaries were then left with participants to complete over the next two weeks. Each participant was reminded by regular phone calls or text messages to complete the diary. Field workers then re-visited after 14 days to review diaries, clarify any unclear entries, and collect the completed diaries. Participants were given in-kind reimbursements (food baskets or mobile phone credit) for their time and the inconvenience of filling out the diary. The initial intent was to run the four two-week periods at regular intervals over one year to capture seasonal differences; however, we faced practical challenges in doing so. These included heavy rains impeding access to the rural study site in Moshi, Tanzania; staffing issues resulting in fieldwork delays in Cape Town and Mount Frere, South Africa; and Zimbabwe’s political and economic crisis resulting in limited cash available for the study team to purchase fuel. As a result, data collection phases were timed to capture seasonal variation where possible and to take account of the feasibility of visiting households on the first and last day of each 2-week data collection period and maintaining contact with participants in between. After completing each round of diary data collection, the expenditure data from the pictorial diaries were double entered into Excel and discrepancies were checked and rectified. The final verified Excel files from each site were imported into Stata 15 for analysis.

### Data analysis

All reported expenditures were converted to monthly per capita amounts and adjusted to a common base year and currency for which exchange rates were available for all included countries (2014 United States Dollars, USD). Outlier observations were top-coded to the value of the 99^th^ percentile to increase the robustness of mean estimates [18]. We calculated the means for each expenditure category and the totals across categories, and used paired t-tests to compare these between the diaries and PURE survey. While the paired t-test is valid to assess these differences given the sufficient country sample sizes, results from the Wilcoxon signed-rank test, a non-parametric alternative to the paired, t-test are presented in S5 Appendix, along with corresponding medians to assess the robustness of our findings.

### Ethics statement

Ethical approval for this study was granted by the Observational Research Ethics Committee at the London School of Hygiene & Tropical Medicine (10622/RR/4295); the Senate Research Committee of the University of the Western Cape (15/4/25); the NWU Health Sciences Ethics Office for Research, Training and Support in South Africa (NWU-00100-16-S1); the National Institute for Medical Research in Tanzania (NIMR/HQ/R.8c/Vol.1/546); and the Medical Research Council of Zimbabwe (MRCZ/A/1722). All participants gave written informed consent.

## Results

The total number of participants who completed at least one phase of data collection is shown in Table 1. While some participants dropped out of the study entirely, others missed one or more phases, but then completed a subsequent phase. The number of participants who completed all four phases was highest in Tanzania (83%) and lowest in Zimbabwe (20%). In South Africa, due to a complete change in research team staff in Cape Town, follow up of 40 original participants was not possible after phase 3, and 40 new participants were recruited to replace them for phase 4 only. The characteristics of participants who did or did not complete all four phases of the diary study are outlined in S6 Appendix. While overall the two groups were similar, in South Africa those who completed 3 phases or fewer were more commonly from urban areas (74%) compared to those who completed all four phases (47%). The opposite was true in Zimbabwe, where these figures were 25% and 45%, respectively.

**Table 1:** Number (and %) of participants completing any phase of diary data collection by country and number of phases completed.

Country	Number of phases completed				Total
	1	2	3	4	
South Africa	44 (13%)	83 (25%)	25 (8%)	177 (54%)	329
Tanzania	11 (4%)	3 (1%)	36 (12%)	251 (83%)	301
Zimbabwe	73 (23%)	59 (19%)	119 (38%)	62 (20%)	313

Table 2 shows the mean per capita monthly household expenditure in 2014 USD reported in the diaries vs. the PURE survey (S7 Appendix provides these figures 2014 local currency units). These figures include data from all participants who completed at least one round of diaries that could be matched to their corresponding PURE expenditure survey data. In all countries, expenditure on food, non-food non-health items, health, and consequently, total household expenditure reported by diaries was consistently higher than that reported by surveys, with all differences statistically significant (p<0.001). The share of total expenditure allocated to health also varied between the survey and diary, from 2% for the survey in each country to 8%, 12%, and 20% for the diary in South Africa, Tanzania, and Zimbabwe.

**Table 2:** Mean monthly household per capita expenditure via PURE survey vs pictorial diary, 2014 USD.

Expenditure category	South Africa (n = 307)			Tanzania (n = 281)			Zimbabwe (n = 294)		
	Survey	Diary	p-value*	Survey	Diary	p-value*	Survey	Diary	p-value*
Total	58.51	153.27	<0.001	32.54	133.58	<0.001	39.61	89.01	<0.001
Food	43.47	78.90	<0.001	21.93	58.75	<0.001	18.77	30.89	<0.001
Non-food non-health	17.39	61.36	<0.001	8.81	59.06	<0.001	19.07	40.56	<0.001
Health	1.00	13.01	<0.001	0.77	15.77	<0.001	0.67	17.57	<0.001

*p-value from 2-sided paired t-test for the difference in means.

**Fig 2:**
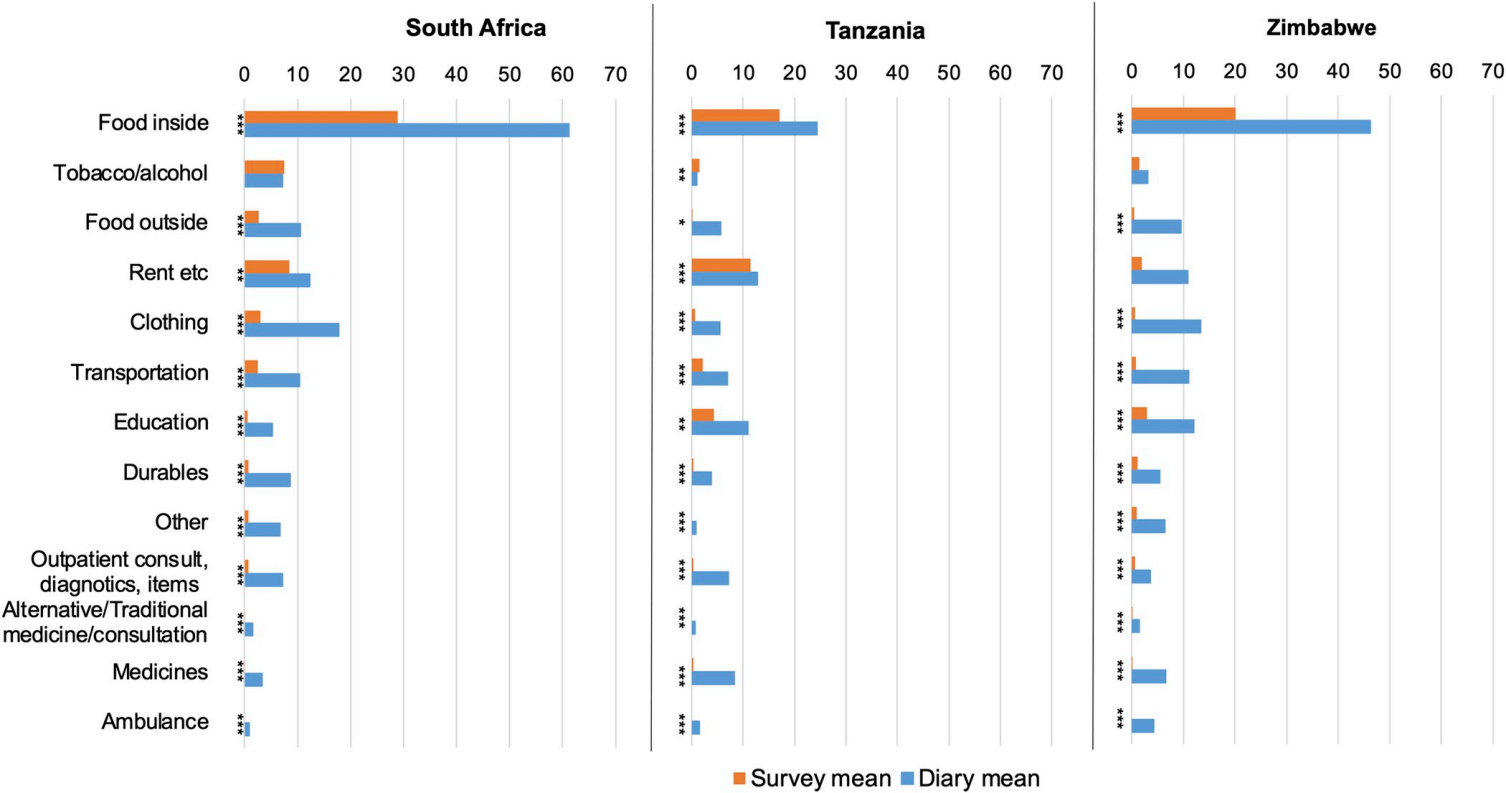
Mean monthly household per capita expenditure by category via PURE survey vs. pictorial diary, 2014 USD. 2-sided paired t-test for the difference in means: * p<0.05, ** p<0.01, *** p<0.001.

A detailed breakdown of expenditure by categories that were included in both the PURE survey and the pictorial diaries showed that diaries consistently capture statistically significantly (at the 5% level) higher estimates for nearly all of the categories (Fig 2). However, the differences were not significant for the category “rent, utilities, phone credit” in Zimbabwe, or for the “tobacco and alcohol” in South Africa or Zimbabwe.

In addition, our diaries included expenditure categories that are typically not included in surveys and were not included in the PURE household expenditure survey (Table 3). We did not include these in our comparisons of totals from surveys vs. diaries above, but analysed them separately. These amounted to a mean of 23.23 USD per capita monthly expenditure in South Africa, 39.23 USD in Tanzania and 21.05 USD in Zimbabwe, accounting for 15%, 29% and 24% of mean total expenditure, by respective country.

**Table 3:** Mean monthly household per capita expenditure on items included in the diaries only, 2014 USD.

Expenditure category	South Africa (n = 307)	Tanzania (n = 281)	Zimbabwe (n = 294)
Family transfer	10.95	8.84	4.21
Farm and garden	1.90	17.55	12.36
Church donation	6.92	6.53	3.21
Caregiver	2.63	4.57	1.08
Religious healing	0.82	1.74	0.19
Total	23.23	39.23	21.05

**Fig 3:**
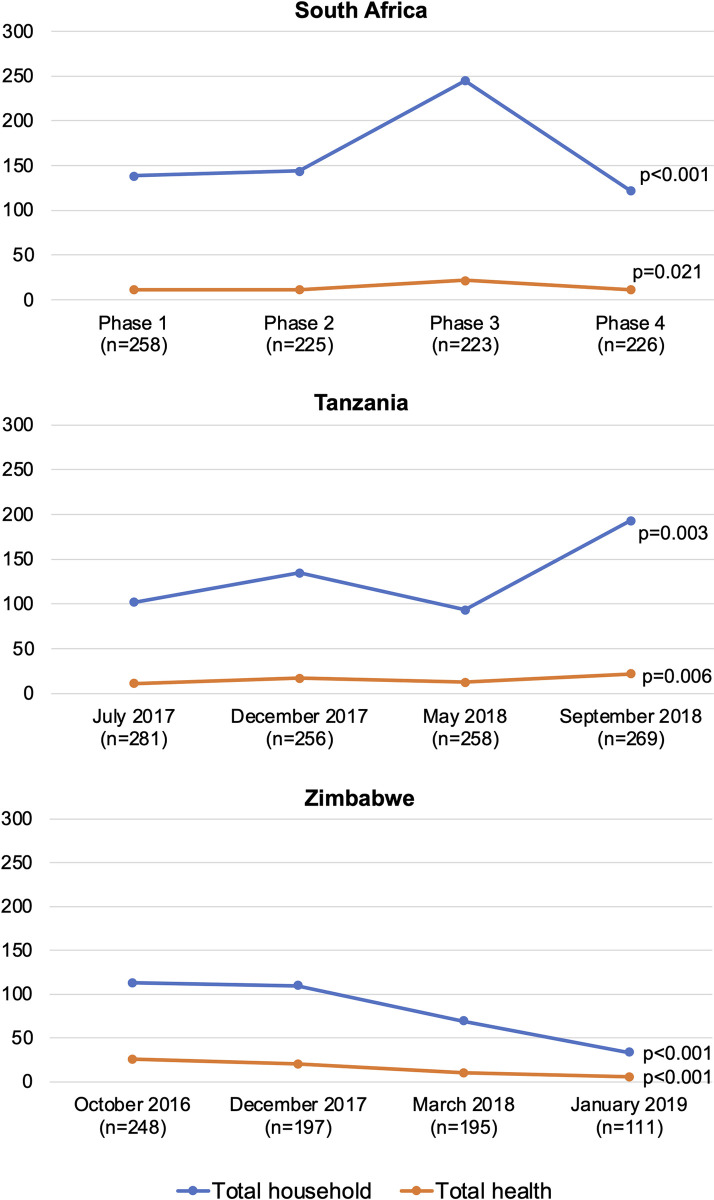
Mean monthly total household and health per capita expenditure by diary data collection phase, 2014 USD. p-values from the ANOVA test for the equality of means across the four phases.

A comparison of data collected in each phase suggests that household expenditure varies by time of year (Fig 3). Results from Tanzania show relatively stable levels of total household spending in the first three phases and an increase in the fourth phase in September 2018. Zimbabwe shows high spending in the pre-Christmas season with lower spending at the beginning of the year, particularly in January 2019. For South Africa, it was not possible to produce a graph with four specific months associated with each of the four phases. There were four different sites in South Africa, and all ran phases at different times. As a result, the graph is presented by the data collection phase (1–4), rather than by month of the year. The level of reported spending appears constant except for Phase 3, where average spending increases. For many, but not all, households phase 3 took place during Spring 2017. As respondents did not always complete all phases of data collection, and in some cases missed a phase but then returned for a later phase, we have indicated the number of diary respondents per phase in each graph.

## Discussion

Pictorial expenditure diaries offer a method of household expenditure data collection that can serve as an alternative to surveys or a means of assessing the extent of bias in surveys. However, few examples have been documented. Our study is novel in that it compares the estimates produced by pictorial diaries to survey estimates from the same study participants. As there is no accepted gold standard for measuring household OOP spending, we can only report on differences between methods. Nevertheless, our findings suggest that the choice of method for collecting data on health expenditure can have significant consequences for expenditure estimates. Specifically, we found that mean monthly expenditure reported in pictorial diaries was higher than that reported in surveys for total overall expenditure, total food expenditure, total non-food non-health expenditure and total health expenditure. This was most dramatically so for health expenditure, with the consequence that the share of total consumption occupied by health expenditure was larger for diaries compared to surveys. It is unlikely that the differences are due to the inclusion of a greater number of expenditure categories in the diaries than in the surveys. We see these differences in expenditure estimates even when considering only those categories that are consistent between the two methods, except for tobacco and alcohol.

The apparent under-reporting of expenditure in retrospective surveys is consistent with previous studies comparing diaries and survey estimates health care utilisation [5] and health-related behaviours [19]. There are several possible reasons why surveys may underestimate health expenditure relative to diaries. First, diaries have the advantage of allowing participants to enter expenditure when it happens and are, thus, less vulnerable to recall biases inherent in retrospective surveys. A second and related point is that, even if the expenditure is not entered as it happens in diaries, diary participants are called regularly by field workers and reminded to fill in the diary. In studies such as ours where field workers called participants weekly, the most extended recall period is in effect a week, compared to typical expenditure survey recall periods of a month. A study in India that allocated households to survey groups with different recall periods found that choice of recall period had a substantial effect on reported health care utilisation, particularly among the poor. The poor reported more health care use with shorter recall periods than the rich; with more extended periods, it was the reverse. The authors hypothesised that as time passes, illness is no longer experienced as an “extraordinary event” but rather a part of regular life, especially by the poor [20]. The same might be said about health care expenditure, perhaps particularly for expenditure on chronic health conditions.

Also, as our surveys were collected at different times of the year, they may have been more likely to pick up periods of high expenditure than surveys conducted at a single point in time. Seasonal variation in expenditure estimates has been well documented [21]. In our case, it is possible that differences in average expenditure reported in diaries used at different times could also be related to macro-level economic factors. The most obvious example is in Zimbabwe, which over the course of our diary data collection experienced a military coup and worsening economic crisis that has greatly negatively affected household incomes and their levels of expenditure. Nonetheless, whether using surveys or diaries, our findings highlight the potential value in repeated visits to households for data collection, to aid in identifying measurement errors like seasonal biases or to understand real variations in expenditure over time.

Importantly, the higher estimates across all categories obtained using data from pictorial diaries may reflect their ability to collect more complete expenditure information from those with low literacy, compared to both surveys and traditional diary approaches [7,13,14]. However, given that diary data was collected from households several years after the survey data, the higher diary estimates, particularly for health-related expenditures, may also be due in part to changes in household composition over time, participant aging and worsening health status over time.

If pictorial diaries can overcome recall and seasonal biases, and the challenges of assessing populations with low literacy, they may offer a gold standard against which surveys can be compared. However, any potential advantage in terms of accuracy provided by diaries must be balanced with practical considerations. In addition to the higher costs of implementing diaries compared to surveys noted in previous work [7], our study highlights the significant challenges of implementing pictorial diaries, particularly if aiming to do so with the same participants at more than one point in time. We experienced high numbers of people not completing all four phases of diary data collection. While the number of participants who did not complete all four phases in Tanzania (17%) was lower than in a previous pictorial diary study in the Gambia (drop-out rate 20–24%) [14], the rates in South Africa and Zimbabwe were significantly higher. Much of this was likely affected by practical challenges and long delays in re-visiting participants for diary collection, including a complete turnover of fieldwork staff in two South African study sites and the inability to access funds for transport in Zimbabwe. However, it may also be related to participant fatigue. While we offered in-kind reimbursement to participants in each country to compensate for the time and inconvenience of completing a paper diary, in Zimbabwe the value of this reimbursement declined over the duration of the study. At the study outset, the value budgeted for reimbursement was sufficient for a large bag of rice and beans. However, as the currency value plummeted due to the financial crisis, the budgeted amount was only sufficient for a bag of rice. Thus, it may not have provided an adequate incentive for participation. Our study also highlights the dependence of long-term diary data collection on field workers and their capacity to visit participants’ homes. In the study by Wiseman et al., field workers visited participants’ homes weekly. While we aimed to do the same, where this was impossible (e.g. due to floods or lack of resources), field workers called participants. They were not always successful with either meeting or reaching participants and often had to make repeat visits or calls.

Another important consideration for a gold standard expenditure tool is its ability to derive valid distributional measures, such as wealth-related inequalities or poverty headcounts, which rely on the ranking or categorisation of households as either richer or poorer. In the absence of a gold-standard against which we can benchmark our findings, we have conducted supplemental analysis comparing how total household expenditure (categorised into tertiles) differs when using diary vs survey data (see S8 Appendix). The two approaches consistently classified 51% of households in South Africa, 43% in both Tanzania and Zimbabwe. The survey approach also appeared to categorise more households as richer than the diary approach in Tanzania; but similar patterns were not observed in the other two study countries. More methodological research is clearly needed to determine the validity and relative benefits of different approaches to collect household expenditure data, particularly in low-income and low-literacy settings.

Finally, we used paper diaries in this study but, with increasing access to mobile phones, network coverage and, in due course, smartphones, there is scope for exploring digital solutions. These have several potential advantages. First, unlike diaries, the multifunctional nature of phones means that they are more likely to be carried at all times, and it is possible to send regular prompts if participants do not engage with them. Second, they come with a low risk of data loss, and data entry can be automated. Thirdly, digital diaries can be implemented using relatively low-tech, low-cost and more ubiquitously accessible SMS text messaging (e.g. a message per diary category per phase) or via voice mail, all of which can be automated or administered live by the researcher. However, they are not a panacea. For example, one study conducted in South East Asia that used SMS-based digital diaries to capture the impact of chronic conditions on daily life found that some participants, especially older people, experienced challenges submitting text message diary entries and were less likely to engage with them [22]. There are also technical issues to be considered, including phone ownership, network coverage and data charges.

## Limitations

We have discussed above the many challenges related to implementing a pictorial diary. These challenges are likely to have had implications on the estimates of expenditure produced by the diaries, compromising their validity. There were high drop out rates in all countries. While those who remained in the study and those who dropped out were overall similar in terms of demographic characteristics, it is possible that those who remained in a given phase were those with higher expenditure at that time and thus more interest or motivation to report it. The 40 new participants recruited in South Africa may have also reported more expenditure as they were new to the study and therefore not vulnerable to respondent fatigue. These limitations should be considered in interpreting our results and in deciding which method to use for expenditure data collection.

## Conclusion

Monitoring household OOP health expenditure globally has become central to efforts to reduce the burdens faced by households due to ill health and to monitor progress toward Universal Health Coverage. However, efforts to do so must consider the limitations of the most commonly used approaches. For example, despite the practical challenges with pictorial expenditure diaries, the difference in estimates produced by diaries and surveys is worrisome, given the dependence on surveys for monitoring household health expenditure. This must be considered together with other limitations of primary measures used to monitor household expenditure previously highlighted. These include the failure of cross-sectional expenditure data collection to consider the long-term economic effect on households of financing strategies such as borrowing money or selling assets [23,24], and the sensitivity of financial protection measures such as catastrophic spending to how expenditure thresholds are defined [25,26].

## Supporting information

S1 Appendix Details on the representativeness of the PURE cohort. (PDF)

S2 Appendix Years of PURE follow-up/out-of-pocket health expenditure data collection by country. (PDF)

S3 Appendix PURE health expenditure questionnaire. (PDF)

S4 Appendix Example pictorial diary tool used in Tanzania. (PDF)

S5 Appendix Per capita median monthly household expenditure via PURE survey vs. pictorial diary, 2014 USD. (PDF)

S6 Appendix Characteristics of participants who did or did not complete all four phases of the diary study. (PDF)

S7 Appendix Mean monthly household per capita expenditure via PURE survey vs pictorial diary, 2014 local currency units. (PDF)

S8 Appendix Comparison of total household expenditure tertiles by data collection method (PURE survey vs pictorial diary) by country, frequencies and (%). (PDF)
