# Supplementary material for: Comparing estimates of household expenditures between pictorial diaries and surveys in three low- and middle-income countries
Source: PLOS Glob Public Health. 2023 Apr 4;3(4):e0001739. doi: 10.1371/journal.pgph.0001739 (PMC10072456; doi:10.1371/journal.pgph.0001739)
Supplement: S1 Appendix — (PDF) [file pgph.0001739.s001.pdf]

S1 Appendix: Details on the representativeness of the PURE cohort

a) Are the countries included in the PURE cohort atypical?

We compared the countries participating in PURE with those participating in MONICA,[1] the largest previous study of this nature conducted. We plotted national income (GDP/capita) against research output (number of publications recorded in SCOPUS between 1996 and 2010 per 100,000 population). These data are taken from a previous study undertaken by one of the authors on global health research capacity to inform policy discussions within WHO. [2] These graphs (Figures S1 and S2) demonstrate that PURE has captured the full diversity of countries on these two dimensions (Figure S1), in marked contrast to MONICA, which was concentrated in high income countries with substantial research capacity (Figure S2).

b) Are PURE populations representative of the countries in which they are situated?

The PURE household population compared to national statistics had more women (sex ratio 95.1 men per 100 women vs 100.3) and was older (33.1 years vs 27.3), although age had a positive linear relationship between the two data sources (Pearson's r = 0.92). PURE was 59.3% urban compared to an average of 63.1% in participating countries. The distribution of education was less than 7% different for each category, although PURE households typically had higher levels of education. For example, 37.8% of PURE household members had completed secondary education compared to 31.3% in the national data. However, age-adjusted annual mortality rates showed positive correlation for men (r = 0.91) and women (r = 0.92) but were lower in PURE compared to national statistics (7.9 per 1000 vs 8.7 for men; 6.7 vs 8.1 for women) (Figure S3). These findings indicate that modest differences exist between the PURE household population and national data for the indicators studied. These modest differences, however, are unlikely to have much influence on exposure-disease (or health systems assessments vs outcome) associations derived in PURE. [3]

Figure S1: Countries Participating in PURE by National Income and Medical Research Capacity

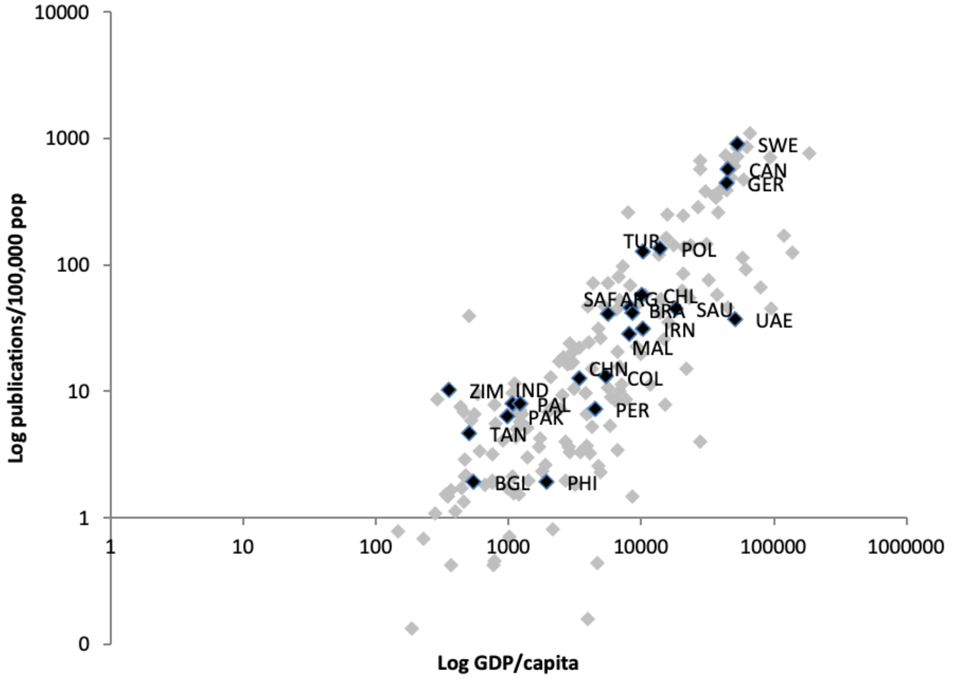

Figure S2: Countries Participating in MONICA by National Income and Medical Research Capacity

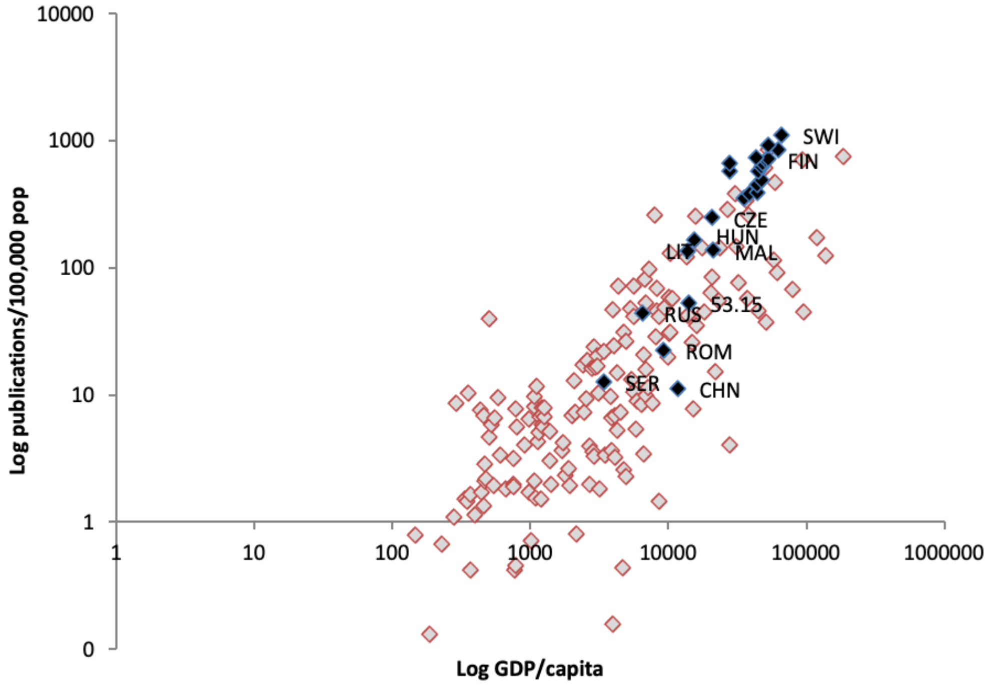

For clarity, only a few of the richest and most productive countries in the MONICA study are labeled in this graph. However, all of the middle-income or lower-income countries, and those with lower levels of research productivity, are labeled.

Figure S3: Mortality in PURE Households Reported in the Previous Two Years Versus National Mortality Data

Male mortality

Female mortality

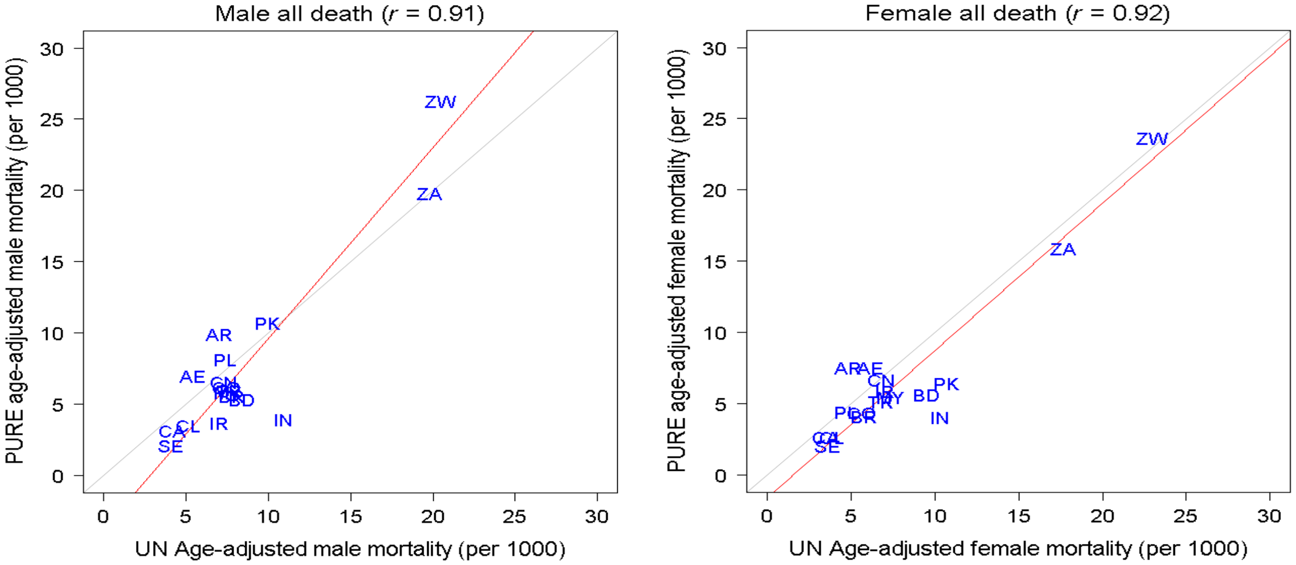

References

1. Tunstall-Pedoe H, Vanuzzo D, Hobbs M, Mahonen M, Cepaitis Z, Kuulasmaa K, et al. Estimation of contribution of changes in coronary care to improving survival, event rates, and coronary heart disease mortality across the WHO MONICA Project populations. Lancet. 2000;355(9205):688-700.
2. McKee M, Stuckler D, Basu S. Where there is no health research: what can be done to fill the global gaps in health research? PLoS Med. 2012;9(4):e1001209. doi: 10.1371/journal.pmed.1001209.
3. Corsi DJ, Subramanian SV, Chow CK, McKee M, Chifamba J, Dagenais G, et al. Prospective Urban Rural Epidemiology (PURE) study: Baseline characteristics of the household sample and comparative analyses with national data in 17 countries. American heart journal. 2013;166(4):636-46.e4. doi: 10.1016/j.ahj.2013.04.019.
