# Supplementary material for: Comparing estimates of household expenditures between pictorial diaries and surveys in three low- and middle-income countries
Source: PLOS Glob Public Health. 2023 Apr 4;3(4):e0001739. doi: 10.1371/journal.pgph.0001739 (PMC10072456; doi:10.1371/journal.pgph.0001739)
Supplement: S2 Appendix — (PDF) [file pgph.0001739.s002.pdf]

S2 Appendix: Years of PURE follow-up/out-of-pocket health expenditure data collection by country

Country	Start date	End date
South Africa	Jan-2014	Sep-2017
Tanzania	Nov-2015	Jun-2018
Zimbabwe	Dec-2014	Jan-2017
