# Supplementary material for: Comparing estimates of household expenditures between pictorial diaries and surveys in three low- and middle-income countries
Source: PLOS Glob Public Health. 2023 Apr 4;3(4):e0001739. doi: 10.1371/journal.pgph.0001739 (PMC10072456; doi:10.1371/journal.pgph.0001739)
Supplement: S3 Appendix — (PDF) [file pgph.0001739.s003.pdf]

S3 Appendix: PURE health expenditure questionnaire

Facing Page Instructions

CRF 161

Income and Expenditure: **Please report ALL income and expenditure in local country currency**

11. Weekly Household Expenditure:

- If any of the listed household expenditures did not occur in the last 7 days, check 'None'.
- If the informant does not know the amount spent for a particular household expenditure OR does not want to disclose this information, check 'Unknown'.

12. Monthly Household Expenditure:

- If any of the listed household expenditures did not occur in the last 30 days, check 'None'.
- If the informant does not know the amount spent for a particular household expenditure OR does not want to disclose this information, check 'Unknown'.

13. Monthly Health Expenditure:

- If any of the listed health expenditures did not occur in the last 30 days OR if the total expense of that health expenditure was reimbursed by insurance, check 'NONE'.
- If the informant does not know the amount spent for health expenditure OR does not want to disclose this information, check 'Unknown'.
- If a part of the health expense was reimbursed by insurance, only indicate the amount NOT reimbursed by insurance (i.e. personally paid).

For e.g., if dental care cost 1000 (**Local currency),** out of which 700 (**Local currency)** was reimbursed by health insurance, indicate 300 (**Local currency)** (1000 minus 700) for 'Question 11e. Dentist or dental care'

Currency Code: Provide the corresponding currency code for all of your weekly, monthly and yearly expenditures. All expenditures must be provided using the SAME currency. Conversion will be required, if you pay for your expenditures with different currencies.

01: ARS, Argentine Peso
02: BDT, Bangladeshi Taka
03: BRL, Brazilian Real
04: CAD, Canadian Dollar
05: CLP, Chilean Peso
06: CNY, Chinese Yuan Renminbi
07: COP, Colombian Peso
08: INR, Indian Rupee
09: IRR, Iranian Rial
10: MYR, Malaysian Ringgit
11: PKR, Pakistani Pupee
12: PLN, Polish Zioty
13: ZAR, South African Rand
14: SEK, Swedish Krona
15: TRY, Turkey Lira
16: AED, Emirate Dirham
17: ZWD, Zimbabwean Dollar
18: ILS, Israeli Shekel
19: TZS, Tanzanian Shilling
20: SAR, Saudi Riyal
21: PHP, Philippine Peso
22: PEN, Peruvian Nuevo Sol
23: EUR, Euro
24: USD, US Dollar

PURE Household Contact Form CRF 161

PURE Household #061 **Plate #161** **Visit #003**

Household ID: ☐☐ (Centre #) ☐☐☐ (Community #) ☐☐☐ (Household #)

Subject Initials ☐☐☐ (F M L)

11. Weekly Household Expenditure: *(See facing page for instructions)*

See facing page
Currency: ☐☐

11. How much was spent in the **last 7 days** on?

		None (No expenditure in last 7 days)	Unknown
11a) Food for the entire household (fruits, vegetables, rice, lentils, meat, milk, eggs spices, oils, sancks etc.)	________	☐	☐
11b) Tobacco, alcoholic beverages and non-alcoholic beverages (tea, coffee, juice, soft drinks etc.) for the entire household	________	☐	☐
11c) Food eaten outside the dwelling (at vendors, kiosks or restaurants)	________	☐	☐

12. Monthly Household Expenditure: *(See facing page for instructions)*

12. How much was spent in the **last 30 days** on?

		None (No expenditure in last 30 days)	Unknown
12a) Rent/mortgage and utilities (electricity, water, cooking/heating fuel telephone/mobile phone, internet, cable tv etc.)	________	☐	☐
12b) Clothing (footware, shirts, pants, coats etc.) and other personal items (soap, shampoo, cosmetics, shaving cream, deodorants etc.)	________	☐	☐
12c) Transportation costs (public transit fares, fuel for personal vehicle etc.)	________	☐	☐
12d) All other goods and services (housekeeping services, laundry supplies, housekeeping supplies, children's toys, pet supplies etc..)	________	☐	☐

13. Monthly Health Expenditure: *(See facing page for instructions)*

Following questions are about how much your household and all its members spent in cash or in-kind on all health care and services that **DID NOT** require an overnight stay for the **last 30 days**. If payment was in-kind, please estimate monetary value. **Please exclude costs to be reimbursed by insurance**

		None	Unknown
13a) Consultation fees by doctors, nurses, that DID NOT require an overnight stay	________	☐	☐
13b) Consultation fees by traditional or alternative healers (Ayurveda, Homeopathy, Chinese medicine etc.)	________	☐	☐
13c) Diagnostic and laboratory tests such as X-rays or blood, urine tests	________	☐	☐
13d) One month supply of medication or drugs (prescription, non-prescription traditional, traditional Chinese, homeopathic etc.)	________	☐	☐
13e) Dentist or dental care	________	☐	☐
13f) Ambulance	________	☐	☐
13g) Any other health care product or services that were not included above? *Please specify:* ________	________	☐	☐

PHRI internal use only ☐☐

Facing Page Instructions

CRF 162

14. Yearly Household Expenditure:

•If any of the listed household expenditures did not occur in the last 12 months, check 'NONE'.

•If the informant does not know the amount spent for a particular household expenditure OR does not want to disclose this information, check 'Unknown'.

PURE **Household Contact Form** **CRF 162**

PURE Household #061 **Plate #162** **Visit #003**

Household ID: ___ ___ (Centre #) ___ ___ ___ (Community #) ___ ___ ___ (Household #)

Subject Initials ___ ___ ___ (F M L)

14 Yearly Household Expenditure: *(See facing page for instructions)*

14. In the **last 12 months,** how much did the family spend on:

			None (No expenditure in last 12 months)	Unknown
14a)	Education fees and supplies (tuition, course fees, books etc.)	______	☐	☐
14b)	Durable goods (televisions, phones, bed sheets, towels, tools etc.) furniture, house appliances (refrigerators, washing machines, micro-wave etc.), vehicles and vehicle upkeep repair	______	☐	☐
14c)	Taxes (property tax, vehicle tax, income tax) and non-health related insurance (personal, vehicle, household, life etc.)	______	☐	☐
14d)	Mandatory health insurance premium, pre-paid health plans or voluntary health insurance premiums (including community health insurance schemes)	______	☐	☐
14e)	Health related items (prescription glasses, hearing aids, canes prosthetic devices etc.) ***Note: Please exclude any reimbursement from insurance***	______	☐	☐
14f)	Costs associated with overnight stays in hospital or health facility ***Note: Please exclude any reimbursement from insurance and transportation costs***	______	☐	☐
14g)	Costs associated with long-term care facility (e.g. old age homes) ***Note: Please exclude any reimbursement from insurance and transportation costs***	______	☐	☐
14h)	All other goods and services (property, land, livestock, cleaning services, repair services, child care services etc.)	______	☐	☐

15. Household Income:

15a. How many members in your household earn money from any source, e.g. employment, pensions, **social assistance** etc.? ___ ___

15b. Current total **monthly** household income after deducting taxes (including employment, pensions social assistance, family support etc.) ______ ☐ Don't know

16. Name of Interviewer: (please print) ______ Last Name ______ First Initial

Date ___ ___ ___ ___ (year) ___ ___ (month) ___ ___ (day)

Apr 23, 2014 Version 3.0 FINAL
