# Supplementary material for: Comparing estimates of household expenditures between pictorial diaries and surveys in three low- and middle-income countries
Source: PLOS Glob Public Health. 2023 Apr 4;3(4):e0001739. doi: 10.1371/journal.pgph.0001739 (PMC10072456; doi:10.1371/journal.pgph.0001739)
Supplement: S4 Appendix — (PDF) [file pgph.0001739.s004.pdf]

S4 Appendix: Example pictorial diary tool used in Tanzania

Participant ID: ________________

Date/Tarehe: ________________

Siku	Kiasi		Kiasi
Chakula cha nyumbani 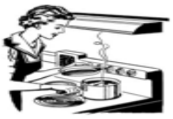		Mavazi 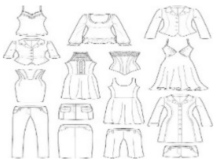	
Chakula kisichokuwa cha nyumbani/Mgahawani/ hotelini 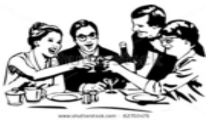		Bidhaa zinazodumu/zitumikazo mfano kununua vocha, kulipa bili ya maji, umeme n.k 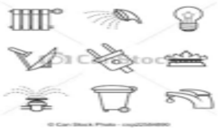	
Elimu mfano kununua madaftari, kupatia nauli za shule n.k 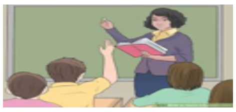		Kaya 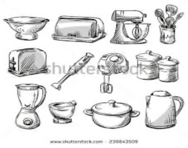	
Usafiri/Usafirishaji 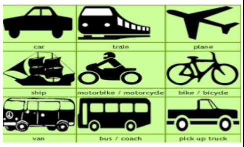		Shamba na bustani 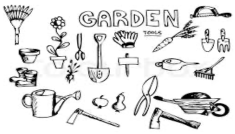	
Pombe na tumbaku 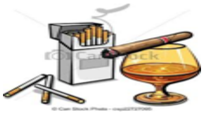		Mchango wa kanisani/ msikitini 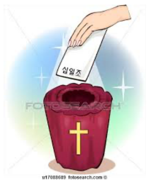	
Mchango kwa familia. 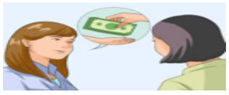		Mchango wa huduma za hospitali/ Kliniki 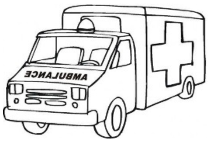	
