# Supplementary material for: Comparing estimates of household expenditures between pictorial diaries and surveys in three low- and middle-income countries
Source: PLOS Glob Public Health. 2023 Apr 4;3(4):e0001739. doi: 10.1371/journal.pgph.0001739 (PMC10072456; doi:10.1371/journal.pgph.0001739)
Supplement: S5 Appendix — (PDF) [file pgph.0001739.s005.pdf]

S5 Appendix: Per capita median monthly household expenditure via PURE survey vs. pictorial diary, 2014 USD

	South Africa (N=307)			Tanzania (N=281)			Zimbabwe (N=294)		
**Per capita monthly expenditure**	**Survey**	**Diary**	**p-value***	**Survey**	**Diary**	**p-value***	**Survey**	**Diary**	**p-value***
Total expenditure (all categories)	37.56	80.91	0.0000	23.32	94.97	0.0000	26.81	71.81	0.0000
Total expenditure (comparable)	36.63	74.12	0.0000	23.16	75.50	0.0000	26.81	58.62	0.0000
Total food (comparable)	23.88	41.36	0.0000	16.89	31.39	0.0000	11.78	22.08	0.0000
Total non-food non-health (comparable)	8.68	21.74	0.0000	3.12	28.40	0.0000	7.47	22.04	0.0000
Total health (comparable)	0.00	2.00	0.0000	0.00	5.90	0.0000	0.00	7.05	0.0000
Food inside	17.04	33.27	0.0000	15.69	25.64	0.0000	11.10	17.45	0.0000
Tobacco/alcohol	2.27	2.00	0.5285	0.00	0.34	0.0000	0.00	0.00	0.0007
Food outside	0.00	3.49	0.0000	0.00	2.47	0.0000	0.00	2.39	0.0000
Rent etc	4.14	4.19	0.3152	0.72	6.54	0.0000	1.32	2.06	0.8220
Clothing	0.00	3.66	0.0000	0.00	5.29	0.0000	0.00	2.04	0.0000
Transportation	0.00	3.86	0.0000	0.00	6.16	0.0000	0.00	3.82	0.0000
Education	0.00	0.69	0.0000	0.35	3.37	0.0000	0.71	2.66	0.0000
Durables	0.00	0.76	0.0000	0.00	1.21	0.0000	0.00	0.60	0.0000
Other	0.00	0.00	0.0000	0.00	1.47	0.0000	0.00	0.00	0.0000
Outpatient consult, diagnostics, items	0.00	0.00	0.0000	0.00	0.72	0.0000	0.00	1.91	0.0000
Alternative/Traditional medicine/consult	0.00	0.00	0.0000	0.00	0.02	0.0000	0.00	0.00	0.0000
Medicines	0.00	0.41	0.0000	0.00	2.25	0.0000	0.00	3.76	0.0000
Ambulance	0.00	0.00	0.0000	0.00	0.81	0.0000	0.00	0.00	0.0000
Dental	0.00			0.00			0.00		
Taxes	0.00			0.00			0.00		
Insurance	0.00			0.00			0.00		
Inpatient stay	0.00			0.00			0.00		
Long term care	0.00			0.00			0.00		
All other yearly (converted to monthly)	0.00			0.00			0.00		
Total of PURE survey-only categories	0.00			0.00			0.00		
Family transfer		0.31			3.25			0.62	
Farm and garden		0.00			4.42			4.88	
Church donation		1.33			2.71			1.39	
Caregiver		0.00			0.00			0.00	
Religious healing		0.00			0.00			0.00	
Total of diary-only categories		4.17			14.70			10.32	

** 2-sided p-value from Wilcoxon rank sum test for difference in medians*
