# Supplementary material for: Comparing estimates of household expenditures between pictorial diaries and surveys in three low- and middle-income countries
Source: PLOS Glob Public Health. 2023 Apr 4;3(4):e0001739. doi: 10.1371/journal.pgph.0001739 (PMC10072456; doi:10.1371/journal.pgph.0001739)
Supplement: S6 Appendix — (PDF) [file pgph.0001739.s006.pdf]

S6 Appendix: Characteristics of participants who did or did not complete all four phases of the diary study

Country	Characteristic	Completed 3 or fewer phases of diary data collection	Completed all 4 phases of diary data collection	p-value^
South Africa	Number of participants	81	226	
	% female	75.3%	77.9%	0.637
	% in urban communities	74.1%	46.9%	0.000
	% with self-reported history of NCD*	51.9%	55.3%	0.592
	Mean size of household	4.4	4.8	0.308
	% with any college or university education	0.0%	1.3%	0.944
	% currently employed	10.1%	10.9%	0.856
Zimbabwe	Number of participants	183	111	
	% female	74.3%	82.0%	0.129
	% in urban communities	25.1%	45.0%	0.000
	% with self-reported history of NCD*	49.2%	57.7%	0.158
	Mean size of household	4.7	5.8	0.133
	% with any college or university education	3.3%	1.8%	0.451
	% currently employed	5.1%	5.6%	0.840
Tanzania	Number of participants	13	268	
	% female	92.3%	79.5%	0.260
	% in urban communities	53.8%	50.0%	0.797
	% with self-reported history of NCD*	53.8%	31.0%	0.088
	Mean size of household	4.3	4.8	0.368
	% with any college or university education	0.0%	0.4%	0.825
	% currently employed	0.0%	4.2%	0.454

**Self-report of being professionally diagnosed with cardiovascular disease, cancer, respiratory disease, kidney disease, diabetes mellitus or hypertension. ^p-value from chi-squared test for difference in percentages or t-test for difference in means*
