# Supplementary material for: Comparing estimates of household expenditures between pictorial diaries and surveys in three low- and middle-income countries
Source: PLOS Glob Public Health. 2023 Apr 4;3(4):e0001739. doi: 10.1371/journal.pgph.0001739 (PMC10072456; doi:10.1371/journal.pgph.0001739)
Supplement: S7 Appendix — (PDF) [file pgph.0001739.s007.pdf]

S7 Appendix: Mean monthly household per capita expenditure via PURE survey vs pictorial diary, 2014 local currency units

	South Africa (N=307)			Tanzania (N=281)			Zimbabwe (N=294)		
**Per capita monthly expenditure**	**Survey**	**Diary**	**p-value***	**Survey**	**Diary**	**p-value***	**Survey**	**Diary**	**p-value***
Total expenditure (all categories)	649.5	1915.5	0.0000	54100.5	285690.3	0.0000	39.7	110.1	0.0000
Total expenditure (comparable)	635.0	1663.4	0.0000	53796.7	220831.1	0.0000	39.6	89.0	0.0000
Total food (comparable)	471.8	856.3	0.0000	36253.3	97126.4	0.0000	18.8	30.9	0.0000
Total non-food non-health (comparable)	188.8	665.9	0.0000	14558.5	97637.4	0.0000	19.1	40.6	0.0000
Total health (comparable)	10.9	141.2	0.0000	1278.1	26067.3	0.0000	0.7	17.6	0.0000
Food inside	313.1	665.0	0.0000	33199.2	76331.7	0.0001	17.0	24.3	0.0001
Tobacco/alcohol	81.7	77.4	0.7093	2286.4	5123.4	0.0058	1.5	1.0	0.1450
Food outside	29.6	113.8	0.0000	582.5	15671.3	0.0123	0.2	5.5	0.0000
Rent etc	91.1	134.4	0.0019	3142.6	17975.2	0.0000	11.5	12.7	0.5317
Clothing	31.7	194.0	0.0000	988.0	22025.2	0.0000	0.6	5.5	0.0000
Transportation	28.0	113.3	0.0000	1253.9	18145.8	0.0000	2.2	6.9	0.0000
Education	7.2	57.0	0.0000	4818.0	19966.3	0.0015	4.3	10.9	0.0000
Durables	8.2	94.3	0.0000	1732.5	8981.5	0.0000	0.3	3.8	0.0000
Other	7.3	73.0	0.0003	1434.0	10543.5	0.0000	0.0	0.8	0.0001
Outpatient consult, diagnostics, items	8.7	77.8	0.0000	1067.8	5952.0	0.0000	0.3	7.2	0.0000
Alternative/Traditional medicine/consult	0.3	17.5	0.0000	7.9	2319.3	0.0001	0.0	0.7	0.0001
Medicines	0.5	35.3	0.0000	76.1	10751.9	0.0000	0.3	8.2	0.0000
Ambulance	0.0	10.6	0.0000	0.0	7044.1	0.0000	0.0	1.5	0.0000
Dental	0.2			0.0			0.0		
Taxes	0.5			78.2			0.0		
Insurance	9.6			10.5			0.0		
Inpatient stay	0.0			27.5			0.0		
Long term care	0.0			0.0			0.0		
All other yearly (converted to monthly)	0.5			84.1			0.0		
Total of PURE survey-only categories	11.3			221.3			0.0		
Family transfer		118.8			14619.2			4.2	
Farm and garden		20.6			29007.7			12.4	
Church donation		75.2			10798.9			3.2	
Caregiver		28.6			7556.3			1.1	
Religious healing		8.9			2877.1			0.2	
Total of diary-only categories		252.1			64859.2			21.1	

** 2-sided p-value from paired t- test for difference in means*
