# Supplementary material for: Comparing estimates of household expenditures between pictorial diaries and surveys in three low- and middle-income countries
Source: PLOS Glob Public Health. 2023 Apr 4;3(4):e0001739. doi: 10.1371/journal.pgph.0001739 (PMC10072456; doi:10.1371/journal.pgph.0001739)
Supplement: S8 Appendix — (PDF) [file pgph.0001739.s008.pdf]

S8 Appendix: Comparison of total household expenditure tertiles by data collection method (PURE survey vs pictorial diary) by country, frequencies and (%)

South Africa	Poorest (survey)	Middle (survey)	Richest (survey)	Total
**Poorest (diary)**	57 (18.6)	37 (12.1)	9 (2.9)	103 (33.6)
**Middle (diary)**	32 (10.4)	38 (12.4)	32 (10.4)	102 (33.2)
**Richest (diary)**	14 (4.6)	27 (8.8)	61 (19.9)	102 (33.2)
**Total**	103 (33.6)	102 (33.2)	102 (33.2)	307 (100.0)

Tanzania	Poorest (survey)	Middle (survey)	Richest (survey)	Total
**Poorest (diary)**	42 (15.0)	41 (14.6)	11 (3.9)	94 (33.5)
**Middle (diary)**	30 (10.7)	30 (10.7)	34 (12.1)	94 (33.5)
**Richest (diary)**	22 (7.8)	23 (8.2)	48 (17.1)	93 (33.1)
**Total**	94 (33.5)	94 (33.5)	93 (33.1)	281 (100.0)

Zimbabwe	Poorest (survey)	Middle (survey)	Richest (survey)	Total
**Poorest (diary)**	43 (14.6)	32 (10.9)	23 (7.8)	98 (33.3)
**Middle (diary)**	32 (10.9)	37 (12.6)	29 (9.9)	98 (33.3)
**Richest (diary)**	23 (7.8)	29 (9.9)	46 (15.7)	98 (33.3)
**Total**	98 (33.3)	98 (33.3)	98 (33.3)	294 (100.0)
